# Ohio State Dental Society

**Published:** 1893-01

**Authors:** 


### Ohio State Dental Society.

The annual meeting of this body was held in Columbus, at the Neil House, 6th, 7th and 8th of December. Quite a large number was in attendance, both of the membership and of visitors. Twenty-six new members were added. Several papers —some of which appear in this number of the *Register*—were read upon interesting subjects, which elicited instructive discussion, in which a large number of those present participated.

Every year demonstrates more fully the importance and value of having the routine business of the body done by a Board of Directors.

Drs. Templeton, of Pittsburgh, Pa., Cravens, of Indianapolis, Ind., and Cassidy, of Covington, Ky., were present, and added much to the interest of the occasion by the presentation of papers and taking part in the discussion of the various subjects presented.

Officers for the ensuing year were elected as follows: President, Geo. H. Wilson, of Cleveland; 1st Vice-President, Chas. Welch, of Wilmington; 2d Vice-President, W. H. Todd, of Columbus; Secretary, L. P. Bethel, of Kent; Assistant Secretary, Henry Barnes, of Cleveland; Treasurer, C. I. Keeley, of Hamilton.

The following were elected directors for three years: C. R. Butler, M. H. Fletcher, A. F. Emminger and M. H. Evans.

This was one of the most satisfactory meetings that the society ever held. The work proceeded from the beginning to the end promptly and without any interruption or friction, and all left, feeling that it had been a profitable time.

